# Generalization bounds for a generator-regularized InfoGAN-inspired adversarial objective

**DOI:** 10.3389/frai.2026.1731256

**Published:** 2026-02-20

**Authors:** Mahmud Hasan, Mathias Nthiani Muia, Md Mahmudul Islam

**Affiliations:** 1 Department of Biostatistics, Virginia Commonwealth University, Richmond, VA, United States; 2 Department of Mathematics and Statistics, University of South Alabama, Mobile, AL, United States; 3 Department of Mathematics, The University of Alabama at Birmingham, Birmingham, AL, United States

**Keywords:** generalization error, generative adversarial networks, neural networks, Rademacher complexity, regularization

## Abstract

The Information Maximizing Generative Adversarial Network (InfoGAN) can be formulated as a minimax problem involving a generator and a discriminator, augmented by a mutual information regularization term. Despite strong empirical performance, rigorous generalization guarantees for InfoGAN-type objectives remain limited, particularly when additional structural components are introduced. In this paper, we study an InfoGAN-inspired adversarial framework obtained by removing the latent code component and introducing an explicit regularization term on the generator, yielding an analytically tractable generator-regularized adversarial objective. We establish generalization error bounds by analyzing the gap between empirical and population objective functions using Rademacher complexity arguments for the discriminator, the generator, and their composition. The resulting bounds reveal explicit *n*^−1/2^ and *m*^−1/2^ decay rates with respect to the discriminator and generator sample sizes and clarify the role of the generator regularization parameter. The theory is further specialized to two-layer neural networks with Lipschitz continuous and non-decreasing activation functions, where explicit entropy-based complexity bounds are derived. Experiments on the CIFAR-10 dataset validate the predicted scaling behavior and demonstrate that the generalization gap decreases systematically as sample size increases, highlighting the stabilizing effect of generator regularization. Overall, this work provides one of the first rigorous generalization analyses for an InfoGAN-inspired adversarial objective with explicit generator regularization.

## 1 Introduction

InfoGAN, which stands for Information Maximizing Generative Adversarial Network (Chen et al., 2016), is an expansion of the conventional Generative Adversarial Network (GAN) framework (Goodfellow et al., 2014). InfoGAN's primary objective is to uncover and manage the structured representations inherent in the data it generates. In the realm of GANs, there exist various variants based on statistical properties, such as Conditional GAN (CGAN) as discussed in Mirza and Osindero (2014), the *f*-GAN as explored in Nowozin et al. (2016), and Wasserstein GAN (WGAN). InfoGAN itself has also given rise to variants like Causal InfoGAN, as described in Wu et al. (2019), and Semi-Supervised InfoGAN (ss-InfoGAN) as detailed in Kurutach et al. (2018). These models have been widely adopted due to their flexibility in modeling complex, high-dimensional distributions and their empirical success across a broad range of applications.

InfoGAN has applications similar to vanilla GANs, including data imaging, natural language processing, and medical image analysis (Reed et al., 2016; Zhu et al., 2017; Yi et al., 2019). A recent review of GANs and their applications is provided in Gui et al. (2023). Beyond classical InfoGAN, several recent InfoGAN-inspired models incorporate additional information-theoretic structure and disentanglement mechanisms. For instance, IB-GAN introduces an information bottleneck constraint within GAN training to encourage disentangled representations while remaining partially InfoGAN-like in spirit (Jeon et al., 2025). Similarly, Double InfoGAN extends InfoGAN ideas to contrastive analysis by leveraging InfoGAN-style regularization to separate common vs. salient generative factors (Carton et al., 2024).

Despite their empirical success, the theoretical foundations of GANs and InfoGANs are not well established, and numerous issues related to their theory and training dynamics remain unresolved (Reed et al., 2016; Liang, 2021; Singh et al., 2018). This has motivated a growing body of recent work aimed at improving stability and generalization in adversarial training. For example, CHAIN proposes a Lipschitz-constrained normalization strategy that targets discriminator overfitting in data-limited regimes and supports improved stability and generalization through theoretical analysis (Ni and Koniusz, 2024). Relatedly, VE-cGAN develops a recent generalization framework for conditional GANs using vicinal estimation, addressing challenges such as limited conditional samples and high-dimensional outputs (Jang and Hwang, 2026). In particular, understanding the statistical generalization properties of adversarially trained models remains a central challenge in modern machine learning theory.

A key question in GAN research is how well these models can approximate a target distribution using a limited number of samples. For instance, the authors in Reed et al. (2016) showed that GANs may fail to generalize under standard metrics even with a polynomial number of samples and established generalization bounds based on neural network distance. The work in Zhang et al. (2018) further analyzed neural network distance and expanded upon these findings. The authors in Liang (2021) and Singh et al. (2018) approached the problem from a nonparametric density estimation perspective. These works highlight both the difficulty and importance of developing rigorous learning-theoretic guarantees for adversarial models.

These recent directions reinforce the need for learning-theoretic guarantees for adversarial objectives, particularly when additional regularization or structural modifications are introduced into the generator or discriminator. However, existing results still have notable shortcomings, and the theoretical analysis of InfoGAN remains relatively rare in the literature. In particular, most available results focus on vanilla GAN objectives and do not address the additional structural components introduced by InfoGAN, such as latent codes and mutual information regularization. A natural direction for theoretical investigation is therefore to evaluate the generalization error of InfoGAN-type objectives under generator regularization by comparing the population objective to its empirical counterpart.

We emphasize that the framework studied in this study is *not* classical InfoGAN in its original form. By removing the latent code variable and introducing an explicit generator regularization term, we obtain an InfoGAN-inspired adversarial objective that is analytically tractable for generalization analysis. This modification preserves the adversarial structure of GANs while enabling explicit control of the generator through regularization. Throughout the study, we therefore focus on the generalization behavior of this generator-regularized adversarial model rather than classical InfoGAN with latent codes.

From a statistical learning perspective, the generator regularization term plays a role analogous to penalization in nonparametric estimation, providing capacity control and enabling explicit bounds on the generalization gap. This viewpoint allows us to bridge ideas from empirical process theory and adversarial learning.

GANs differ from classical density estimation methods by implicitly learning the data distribution through an adversarial process between a generator and a discriminator. Let the generator be denoted by *G* with sample size *m* and the discriminator by *D* with sample size *n*, where *D* aims to distinguish between the data distribution *p*_*x*_ and the generator distribution *p*_*z*_. Let *z* be a noise variable distributed according to *p*_*z*_ and *X* denote a real data variable. The generator transforms noise samples into synthetic data points, while the discriminator attempts to distinguish these generated samples from real observations. Consider GAN models in which both the generator and discriminator function classes are parameterized. The minimax problem of GAN introduced in Goodfellow et al. (2014) can be written as


$$\begin{array}{l} d\left(D,G\right)=\underset{G}{\min}\underset{D}{\max}\left[{𝔼}_{p_{x}}\left[\log D\left(x\right)\right]+{𝔼}_{p_{z}}\left[1-\log D\left(G\left(z\right)\right)\right]\right]. \end{array}$$ (1)

The InfoGAN framework extends this setup by dividing the noise variable *z* into an incompressible noise component and a latent code *c*, so that the generator takes the form *G*(*z, c*). The InfoGAN objective (Chen et al., 2016) is given by


$$\begin{array}{l} d_{I}(D,G)=\underset{G}{\min}\underset{D}{\max}[{𝔼}_{p_{x}}[\log D(x)]+\\ {𝔼}_{p_{z}}[1-\log D(G(z))]-\lambda I(c;G(z,c))], \end{array}$$ (2)

where *I*(*c*; *G*(*z, c*)) = *H*(*c*)−*H*(*c*|*G*(*z, c*)) denotes the mutual information between the latent code and the generated sample, and λ ≥ 0 is a regularization parameter. The mutual information term encourages the generator to encode interpretable structure in the latent variables. However, optimizing *I*(*c*; *G*(*z, c*)) is difficult since it requires the posterior distribution *P*(*c*|*x*).

To address this, a lower bound *L*_*I*_(*c*; *Q*) is introduced by defining an auxiliary distribution *Q*(*c*|*x*) to approximate *P*(*c*|*x*). The practical InfoGAN objective is therefore written as


$$\begin{array}{r} d_{I}\left(D,G\right)=\\ \underset{G}{\min}\underset{D}{\max}\left[{𝔼}_{p_{x}}\left[\log D\left(x\right)\right]+{𝔼}_{p_{z}}\left[1-\log D\left(G\left(z\right)\right)\right]-\lambda L_{I}\left(c;Q\right)\right]. \end{array}$$ (3)

While Equation 3 serves as the primary objective function commonly used in applications, this study opts to consider and subsequently employ Equation 2 as the core objective function for its theoretical analysis. This choice allows us to isolate the effect of generator regularization and to derive explicit learning-theoretic guarantees. This objective function introduces regularization in the generator variable, a departure from the majority of existing literature, which typically lacks such regularization.

The existing theoretical research is primarily based on vanilla GAN error analysis defined by the difference between empirical and population objectives, as in Liang (2021), Huang et al. (2022), Ji et al. (2021), and Zhang et al. (2018). A preprint of this work has previously been published in Hasan and Muia (2025). In this study, the objective function (Equation 2) is used to study generalization properties for an InfoGAN-inspired framework without latent variable *c* in the setting of two-layer neural networks. In this work, we deliberately exclude the latent code variable *c* in order to focus on a generator-regularized adversarial objective that admits explicit generalization analysis. This choice is motivated by analytical tractability rather than by the representational goals of classical InfoGAN. We stress that our results do not apply to classical InfoGAN with latent codes and variational mutual information terms. Moreover, the logarithmic function satisfies log*x* → −∞ as *x* → 0, which may lead to instability in practice. We therefore develop a new objective function without a latent code and with a stable measuring function. The generalization error is defined as the difference between the population version of the objective function and its empirical counterpart. Our analysis quantifies this difference using tools from empirical process theory. The difference between the population and empirical objective functions is bounded using Rademacher complexity. The resulting bounds are derived explicitly for two-layer networks under Lipschitz and non-decreasing activation functions.

Our contributions are threefold: (i) we formulate a generator-regularized, InfoGAN-inspired adversarial objective (without latent code) and cast it as a neural network distance with an explicit generator penalty; (ii) we bound the empirical–population objective gap using Rademacher complexity for the discriminator, generator, and their composition; (iii) we specialize the bounds to two-layer networks under Lipschitz and non-decreasing activations and validate the predicted trends empirically. A concise comparison between classical InfoGAN and the generator-regularized objective studied in this paper is provided in Table 1. The main theoretical contributions and organization of the study are summarized as follows:

- Section 2 presents the derivation of a regularized objective function from InfoGAN, excluding the latent code.
- Section 3 demonstrates that the difference between the empirical and population objective functions is bounded by the Rademacher complexity of the discriminator, generator, and their composition.
- Section 4 formulates the discriminator and generator classes for a two-layer network. The corresponding weight parameters of the network are constrained by constants.
- Section 4 derives upper bounds for the Rademacher complexities in two cases: 1-Lipschitz and non-decreasing activation functions. These bounds are then applied to establish rates for the objective function differences as functions of the discriminator and generator sample sizes.
- Section 5 provides concluding remarks and directions for future research.

**Table 1:** Classical InfoGAN vs. the InfoGAN-inspired generator-regularized objective studied here.

**Aspect**	**Classical InfoGAN (Chen et al., 2016)**	**This paper (InfoGAN-inspired)**
Latent code *c*	Present	Removed (*c* absent/fixed)
Extra term	−λ*I*(*c*; *G*(*z, c*))	−λ*𝔼ϕ*(*G*(*z*)) (generator regularization)
Practical objective	Uses auxiliary *Q*(*c*|*x*)	No latent inference required
Goal	Interpretable representations	Generalization of regularized objective
Theory focus	Limited	Rademacher generalization bounds

Our theory is developed for bounded two-layer networks and an objective without latent codes; Section 6 discusses the implications of these assumptions and directions toward deeper architectures and classical InfoGAN settings.

## 2 Objective function without latent code

In the original InfoGAN framework, instead of using a single unstructured noise vector *z*, the authors divide the input noise vector into two components: an incompressible noise variable, still denoted by *z*, and a latent code denoted by *c*. The generator is trained adversarially to confuse the discriminator while simultaneously maximizing the mutual information between the latent code and the generated samples. This additional structure is intended to encourage the emergence of interpretable and disentangled representations in the generated data.

In this work, we focus on a simplified yet analytically tractable setting by excluding the latent code variable. Specifically, we consider the case in which the latent code is absent and effectively set *c* = 0. This modification allows us to isolate the effect of generator regularization and to derive explicit generalization bounds without the additional complexity introduced by latent-variable inference.

From a theoretical standpoint, removing the latent code eliminates the need to handle variational approximations of mutual information, thereby enabling a direct empirical process analysis of the adversarial objective.

Throughout this section, we assume that the generator output *G*(*z*) admits a density with respect to Lebesgue measure, is bounded, and satisfies *G*(*z*) ∈ [0, 1] almost surely. Under these assumptions, the entropy and expectation terms involving log*G*(*z*) are well-defined and finite. These regularity conditions ensure that all subsequent expectations and entropy terms are mathematically well-posed. Under this setting, Equation 2 reduces to:


$$\begin{array}{l} d_{I}(D,G)=\underset{G}{\min}\underset{D}{\max}[{𝔼}_{p_{x}}[\log D(x)]+{𝔼}_{p_{z}}[1-\log D(G(z))]-\\ \;\lambda I(0;G(z,0))]\\ \;=\underset{G}{\min}\underset{D}{\max}[{𝔼}_{p_{x}}[\log D(x)]+{𝔼}_{p_{z}}[1-\log D(G(z))]\\ \;-\lambda H(0)+\lambda H(G(z,0))]\\ \;=\underset{G}{\min}\underset{D}{\max}[{𝔼}_{p_{x}}[\log D(x)]+{𝔼}_{p_{z}}[1-\log D(G(z))]+\\ \;\lambda H(0|G(z,0))]\\ \;=\underset{G}{\min}\underset{D}{\max}[{𝔼}_{p_{x}}[\log D(x)]+{𝔼}_{p_{z}}[1-\log D(G(z))]+\\ \;\lambda H(G(z))]\\ \;=\underset{G}{\min}\underset{D}{\max}[{𝔼}_{p_{x}}[\log D(x)]+{𝔼}_{p_{z}}[1-\log D(G(z))]-\\ \;\lambda {𝔼}_{p_{z}}\log[G(z)]]. \end{array}$$ (4)

Here, mutual information can be represented equivalently as *I*(0;*G*(*z*, 0)) = *H*(0)−*H*(0|*G*(*z*, 0)), where *H* denotes entropy. Equation 4 presents the objective function with generator regularization in the case where the latent code is zero. In this formulation, the regularization term acts directly on the generator distribution, penalizing low-entropy or degenerate outputs. Under the density and boundedness assumptions stated above, the differential entropy of *G*(*z*) satisfies *H*(*G*(*z*)) = −𝔼_*p*_*z*__log*p*_*G*_(*G*(*z*)), where *p*_*G*_ is the density of *G*(*z*). In our simplified setting, we use a bounded surrogate regularizer of the form −𝔼_*p*_*z*__log(*G*(*z*)) to obtain an analytically tractable generator penalty; replacing log by ϕ in Equation 5 yields a stable objective compatible with integral probability metric analyses. This surrogate regularization can be viewed as a tractable proxy for entropy control on the generator output. However, this can lead to issues in practice, as log*x* → −∞ as *x* → 0. Such behavior may cause numerical instability and poor gradient behavior during optimization. By replacing log with a monotone function ϕ:[0, 1] → ℝ, the objective becomes:


$$\begin{array}{r} d_{I}\left(D,G\right)\\ =\underset{G}{\min}\underset{D}{\max}\left[{𝔼}_{p_{x}}\left[\phi D\left(x\right)\right]+{𝔼}_{p_{z}}\left[1-\phi D\left(G\left(z\right)\right)\right]-\lambda {𝔼}_{p_{z}}\phi \left[G\left(z\right)\right]\right]. \end{array}$$ (5)

**Assumption 1 (Measuring function ϕ)**. Throughout, the measuring function ϕ:[0, 1] → ℝ is assumed to be non-decreasing and *L*_ϕ_-Lipschitz. This ensures that ϕ ∘ *D* remains uniformly bounded and allows standard contraction arguments in the Rademacher analysis.

The replacement of the logarithmic function by a general monotone measuring function ϕ is motivated by both theoretical and practical considerations. In particular, the logarithmic function becomes unstable near zero, while monotone functions allow the objective to be interpreted within the neural network distance and integral probability metric frameworks commonly used in GAN theory. From a learning-theoretic perspective, this replacement also facilitates the use of contraction inequalities and simplifies the derivation of complexity bounds. Here, ϕ is a non-decreasing Lipschitz measuring function (Assumption 1). This can also be written as Reed et al. (2016):


$$\begin{array}{l} d_{I}(D,G)=\underset{G}{\min}\;\underset{D}{\max}[{𝔼}_{p_{x}}[\phi D(x)]+{𝔼}_{p_{z}}[1-\phi D(G(z))]-\\ \;\lambda {𝔼}_{p_{z}}\phi [G(z)]-2\phi (1/2)]. \end{array}$$ (6)

For ϕ(*x*) = *x*, the final objective function with changing the notations becomes:


$$\begin{array}{l} d_{I}\left(D,G\right)=\underset{G}{\min}\underset{D}{\max}\left[{𝔼}_{p_{x}}\left[D\left(x\right)\right]-{𝔼}_{p_{z}}\left[D\left(G\left(z\right)\right)\right]-\lambda {𝔼}_{p_{z}}G\left(z\right)\right]. \end{array}$$ (7)

Equation 7 can be interpreted as a neural network distance augmented with an explicit generator regularization term. This formulation is consistent with existing generalization analyses of GANs based on integral probability metrics and neural network distances, while introducing additional control over the generator through regularization. In particular, it fits naturally within the framework of integral probability metrics with a penalized generator class.

Equation 7 therefore represents a generator-regularized neural network distance. While regularization could in principle be applied to either the discriminator or the generator, we emphasize that in the absence of a latent code variable, the regularization term naturally acts on the generator. This choice is also aligned with the role of the generator as the primary source of model complexity in adversarial learning. Consequently, the regularized objective function in Equation 7 is particularly suitable for adversarial models in which the generator takes an unstructured noise variable as input.

Suppose that ${\left\{X_{i}\right\}}_{i=1}^{n}$ are independent and identically distributed observations drawn from the data distribution *p*_*x*_, and that the generator produces ${\left\{G\left(z_{j}\right)\right\}}_{j=1}^{m}$ as independent and identically distributed samples drawn from the model distribution *p*_*z*_. We assume throughout that the data sample and the noise sample are independent.

We define the two empirical loss functions as follows, based on Equation 7:


$$\begin{array}{l} d_{I}\left(\hat{D},Ĝ\right)=\underset{G}{\min}\underset{D}{\max}\left[\frac{1}{n}\overset{n}{\underset{i=1}{\sum }}D\left(x_{i}\right)-\frac{1}{m}\overset{m}{\underset{j=1}{\sum }}D\left(G\left(z_{j}\right)\right)-\lambda \frac{1}{m}\overset{m}{\underset{j=1}{\sum }}G\left(z_{j}\right)\right]. \end{array}$$ (8)

and


$$\begin{array}{l} d_{I}\left(\hat{D},G\right)=\underset{G}{\min}\underset{D}{\max}\left[\frac{1}{n}\overset{n}{\underset{i=1}{\sum }}D\left(x_{i}\right)-{𝔼}_{p_{z}}\left[D\left(G\left(z\right)\right)\right]-\lambda {𝔼}_{p_{z}}G\left(z\right)\right]. \end{array}$$ (9)

Equation 8 is the fully empirical objective (empirical averages over both the data sample and the noise sample), whereas Equation 9 is a mixed empirical–population objective (empirical average over the data sample and population expectations over the noise distribution). These two formulations will be used to quantify different sources of statistical error in the subsequent generalization analysis. Here, *D*(*G*(*z*)) = *D* ∘ *G* is the composition of the discriminator and generator.

**Notation**

- *n*: number of real data samples *x*_1_, …, *x*_*n*_ ~ *p*_*x*_.
- *m*: number of noise samples *z*_1_, …, *z*_*m*_ ~ *p*_*z*_ used to produce *G*(*z*_*j*_).
- *D*: discriminator function class; *G*: generator function class.
- *D* ∘ *G*: = {*x* ↦ *D*(*G*(*x*)):*D* ∈ *D, G* ∈ *G*} (composition class).
- *Q*_*x*_: uniform bound on discriminator outputs, ||*D*||_∞_ ≤ *Q*_*x*_.
- *Q*_*z*_: uniform bound on generator outputs, ||*G*||_∞_ ≤ *Q*_*z*_.
- $R_{n}\left(\cdot \right)$: empirical Rademacher complexity on *n* samples.
- λ ≥ 0: generator regularization coefficient.

## 3 Bound of objective function difference

The generalization bound of InfoGAN is defined by the difference between the empirical and population versions of the objective function. In particular, we consider the discrepancies between the empirical objective in Equation 8 and its population counterpart in Equation 7, and between the mixed empirical–population objective in Equation 9 and its population counterpart in Equation 7. Considering $\hat{D}$ and Ĝ as the empirical counterparts of *D* and *G*, respectively, the difference in the objective function can be represented as:


$$\begin{array}{l} d_{I}\left(\hat{D},Ĝ\right)-d_{I}\left(D,G\right) \end{array}$$ (10)

$$\begin{array}{l} d_{I}\left(\hat{D},G\right)-d_{I}\left(D,G\right) \end{array}$$ (11)

In Equation 10, this indicates the difference between the empirical objective (based on both samples) and the population objective. Meanwhile, Equation 11 compares the mixed empirical–population objective with the population objective. The subsequent theorem establishes bounds for Equations 10, 11, assuming that both the discriminator *D* and generator *G* are uniformly bounded. The proof employs the Cauchy-Schwarz inequality and McDiarmid's inequality. Throughout, we assume that the data sample ${\left\{x_{i}\right\}}_{i=1}^{n}$ and the noise sample ${\left\{z_{j}\right\}}_{j=1}^{m}$ are independent, and that each sample is i.i.d. from its respective distribution.

**Notation**

Recall that *n* denotes the sample size for *x*_1_, …, *x*_*n*_ ~ *p*_*x*_, and *m* denotes the sample size for *z*_1_, …, *z*_*m*_ ~ *p*_*z*_. We write $R_{n}\left(D\right)$ for the (expected) Rademacher complexity of the discriminator class evaluated on *n* samples from *p*_*x*_, and $R_{m}\left(D\circ G\right)$ for the complexity of the composed class {D∘G:D∈D,G∈G} evaluated on *m* noise samples from *p*_*z*_. For clarity, $R_{n}\left(\cdot \right)$ denotes the (expected) Rademacher complexity, i.e., the expectation is taken over both the sample and the Rademacher signs.

**Theorem 3.1**. *Suppose the sets of discriminator functions *D* and generator functions *G* are symmetric with ∥*f*∥_∞_ ≤ ℚ_*x*_ for all *f* ∈ *D* and ∥*g*∥_∞_ ≤ ℚ_*z*_ for all *g* ∈ *G*. Then, with probability at least 1 − 2δ over the random training samples ${\left\{x_{i}\right\}}_{i=1}^{n}$ and ${\left\{z_{j}\right\}}_{j=1}^{m}$, we have*


$$\begin{array}{r} d_{I}\left(\hat{D},Ĝ\right)-d_{I}\left(D,G\right)\leq 2R_{n}\left(D\right)+2R_{m}\left(D\circ G\right)+2\lambda R_{m}\left(G\right)\\ +2{\mathbb{Q}}_{x}\sqrt{\frac{\log\left(1/\delta \right)}{2n}}+2{\mathbb{Q}}_{z}\left(1+\lambda \right)\sqrt{\frac{\log\left(1/\delta \right)}{2m}} \end{array}$$ (12)

*and*


$$\begin{array}{l} d_{I}\left(\hat{D},G\right)-d_{I}\left(D,G\right)\leq 2R_{n}\left(D\right)+2{\mathbb{Q}}_{x}\sqrt{\frac{\log\left(1/\delta \right)}{2n}}. \end{array}$$ (13)

*Proof*: We first bound Equation 10. Using the definition of Equations 7, 8 and the standard inequality


$$\underset{a}{\sup}F\left(a\right)-\underset{a}{\sup}G\left(a\right)\leq \underset{a}{\sup}(F\left(a\right)-G\left(a\right)),$$

we obtain


$$\begin{array}{l} d_{I}\left(\hat{D},Ĝ\right)-d_{I}\left(D,G\right)\\ =\underset{D\in D}{\sup}\left[\frac{1}{n}\overset{n}{\underset{i=1}{\sum }}D\left(x_{i}\right)-\frac{1}{m}\overset{m}{\underset{j=1}{\sum }}D\left(G\left(z_{j}\right)\right)-\lambda \frac{1}{m}\overset{m}{\underset{j=1}{\sum }}G\left(z_{j}\right)\right]\\ \;-\underset{D\in D}{\sup}\left[{𝔼}_{p_{x}}D\left(x\right)-{𝔼}_{p_{z}}D\left(G\left(z\right)\right)-\lambda {𝔼}_{p_{z}}G\left(z\right)\right]\\ \leq \underset{D\in D}{\sup}\left[\frac{1}{n}\overset{n}{\underset{i=1}{\sum }}D\left(x_{i}\right)-{𝔼}_{p_{x}}D\left(x\right)\right]\\ \;+\underset{D\in D,G\in G}{\sup}\left[{𝔼}_{p_{z}}D\left(G\left(z\right)\right)-\frac{1}{m}\overset{m}{\underset{j=1}{\sum }}D\left(G\left(z_{j}\right)\right)\right]\\ \;+\lambda \underset{G\in G}{\sup}\left[{𝔼}_{p_{z}}G\left(z\right)-\frac{1}{m}\overset{m}{\underset{j=1}{\sum }}G\left(z_{j}\right)\right]. \end{array}$$ (14)

The second term is taken over the composed class *D* ∘ *G*, and the third term is taken over *G* because the generator regularization term does not involve *D*.

We now bound each term using standard symmetrization and McDiarmid/Hoeffding-type concentration; see, e.g., Theorem 3.1 in Zhang et al. (2018) for this template. For completeness, we note that the bounds below follow from (i) symmetrization, (ii) the Rademacher contraction principle for bounded function classes, and (iii) McDiarmid's inequality (or Hoeffding's inequality) applied to bounded differences.

(i) Discriminator term

With probability at least 1 − δ,


$$\begin{array}{l} \underset{D\in D}{\sup}\left[\frac{1}{n}\overset{n}{\underset{i=1}{\sum }}D\left(x_{i}\right)-{𝔼}_{p_{x}}D\left(x\right)\right]\leq 2R_{n}\left(D\right)+2{\mathbb{Q}}_{x}\sqrt{\frac{\log\left(1/\delta \right)}{2n}}. \end{array}$$ (15)

(ii) Composition term

With probability at least 1 − δ,


$$\begin{array}{r} \underset{D\in D,G\in G}{\sup}\left[{𝔼}_{p_{z}}D\left(G\left(z\right)\right)-\frac{1}{m}\overset{m}{\underset{j=1}{\sum }}D\left(G\left(z_{j}\right)\right)\right]\leq 2R_{m}\left(D\circ G\right)+\\ 2{\mathbb{Q}}_{z}\sqrt{\frac{\log\left(1/\delta \right)}{2m}}. \end{array}$$ (16)

Here we use that for any *D* ∈ *D* and *G* ∈ *G*, the composition *D* ∘ *G* is uniformly bounded by ||*D*||_∞_ ≤ *Q*_*x*_, and we absorb constants into *Q*_*z*_ for notational simplicity (as in Section 4).

(iii) Generator regularization term With probability at least 1 − δ,


$$\begin{array}{l} \underset{G\in G}{\sup}\left[{𝔼}_{p_{z}}G\left(z\right)-\frac{1}{m}\overset{m}{\underset{j=1}{\sum }}G\left(z_{j}\right)\right]\leq 2R_{m}\left(G\right)+2{\mathbb{Q}}_{z}\sqrt{\frac{\log\left(1/\delta \right)}{2m}}. \end{array}$$ (17)

Finally, combining Equations 15, 16, 17 into Equation 14, and applying a union bound over the three events (absorbing constants so that the final probability is at least 1 − 2δ), yields


$$\begin{array}{r} d_{I}\left(\hat{D},Ĝ\right)-d_{I}\left(D,G\right)\leq 2R_{n}\left(D\right)+2R_{m}\left(D\circ G\right)+2\lambda R_{m}\left(G\right)\\ +2{\mathbb{Q}}_{x}\sqrt{\frac{\log\left(1/\delta \right)}{2n}}+2{\mathbb{Q}}_{z}\left(1+\lambda \right)\sqrt{\frac{\log\left(1/\delta \right)}{2m}}, \end{array}$$

which is Equation 12.

The bound Equation 13 follows directly from Equation 15, since $d_{I}\left(\hat{D},G\right)$ differs from *d*_*I*_(*D, G*) only through the empirical approximation of 𝔼_*p*_*x*__*D*(*x*). In particular, the generator-related terms remain at their population values in Equation 9, so only the discriminator sampling error contributes to Equation 11.

**Remark 3.1**. *The generalization bound in Theorem 3.1 decomposes the gap between the empirical and population objectives into (i) *complexity* terms, measured by Rademacher complexities, and (ii) *finite-sample* concentration terms, controlled by the uniform bounds and the sample sizes*.

*The term $2R_{n}\left(D\right)$ captures the statistical complexity of the discriminator class when evaluated on the data sample ${\left\{x_{i}\right\}}_{i=1}^{n}$*.

*The term $2R_{m}\left(D\circ G\right)$ measures the complexity of the composed class *D* ∘ *G* when evaluated on the noise sample ${\left\{z_{j}\right\}}_{j=1}^{m}$ through the generated points *G*(*z*_*j*_)*.

*The additional term $2\lambda R_{m}\left(G\right)$ arises from the generator regularization component in the objective. Since the regularization term depends only on *G*, its empirical population deviation is controlled by the Rademacher complexity of the generator class itself*.

*Finally, the remaining terms $2Q_{x}\sqrt{\frac{\log\left(1/\delta \right)}{2n}}$ and $2Q_{z}\left(1+\lambda \right)\sqrt{\frac{\log\left(1/\delta \right)}{2m}}$ arise from concentration of empirical means around expectations under the uniform boundedness assumptions*.

*Overall, Theorem 3.1 shows that the empirical objective approaches its population counterpart as the sample sizes grow and as the effective complexities of *D*, *D* ∘ *G*, and *G* are controlled. In particular, the bound makes explicit how two sources of sampling error contribute separately: the data-sampling error scales with *n* through $R_{n}\left(D\right)$ and the concentration term, while the noise-sampling error scales with *m* through $R_{m}\left(D\circ G\right)$, $R_{m}\left(G\right)$, and the corresponding concentration term. Moreover, the regularization strength λ amplifies the generator-only terms, reflecting a natural bias–variance trade-off: larger λ increases the contribution of $R_{m}\left(G\right)$ and the *m*-dependent concentration term, while potentially improving stability and controlling generator outputs*.

## 4 Application in a two-layer network

This section instantiates the general generalization bounds in Theorem 3.1 for concrete two-layer (one-hidden-layer) neural network classes. We (i) define discriminator and generator hypothesis classes with explicit ℓ_1_-type constraints that control capacity, (ii) bound $R_{n}\left(D\right)$, $R_{m}\left(G\right)$, and $R_{m}\left(D\circ G\right)$ using covering numbers and Dudley-type entropy integrals, and (iii) plug these bounds into Equations 12, 13 to obtain explicit rates in *n* and *m* under two common activation assumptions: Lipschitz and non-decreasing.

The derived bounds in Theorem 3.1 provide valuable insights when applying the infoGAN framework in Equation 7 to a two-layer neural network architecture. In this section, we discuss how these bounds can be useful in analyzing and improving the performance of such networks. The goal is to minimize the objective function disparity between the empirical distributions of $\hat{D}$ and Ĝ, as well as the objective function difference between $\hat{D}$ and *G*. The derived bounds, as shown in Equations 12, 13, provide upper limits on the disparity and difference in the objective functions, respectively. These bounds allow us to assess the potential deviation between the empirical and true objective functions. Furthermore, the analysis of these bounds offers insights into the convergence behavior of the two-layer network. In this section, we will focus solely on the theoretical framework of two-layer neural networks. The applications of a two-layer neural network for the readers can be found in the recent studies by Wang et al. (2019) and Nian and Yao (2018). Our emphasis is on explicit learnability guarantees: we quantify how sampling error decays as *n* and *m* increase, and how architectural constraints (through *V* and the activation choice) control the effective complexity of the adversarial objective.

It is important to note that neural network classes are typically infinite, so bounds involving finite cardinalities such as log|*D*| or log|*G*| are generally not appropriate. In this section, we therefore derive all Rademacher complexity bounds using covering numbers and entropy integrals (Dudley-type bounds), which are standard tools for infinite hypothesis classes.

### 4.1 Mapping to a standard two-layer fully-connected network

A standard two-layer (one-hidden-layer) fully-connected network can be written as *f*(*u*) = *W*_2_*s*(*W*_1_*u* + *b*_1_) + *b*_2_, where *W*_1_ and *W*_2_ are weight matrices, *b*_1_, *b*_2_ are bias vectors, and *s*(·) is an activation function applied elementwise. Our classes *D* and *G* in Equations 19–21 correspond to such networks with ℓ_1_-type constraints on the first-layer weights and bounded second-layer coefficients, ensuring uniform control of network capacity. These ℓ_1_-type constraints are standard in statistical learning theory because they yield tractable entropy bounds and, consequently, explicit Rademacher complexity rates.

### 4.2 Formation of two-layer network

**Figure 1:**
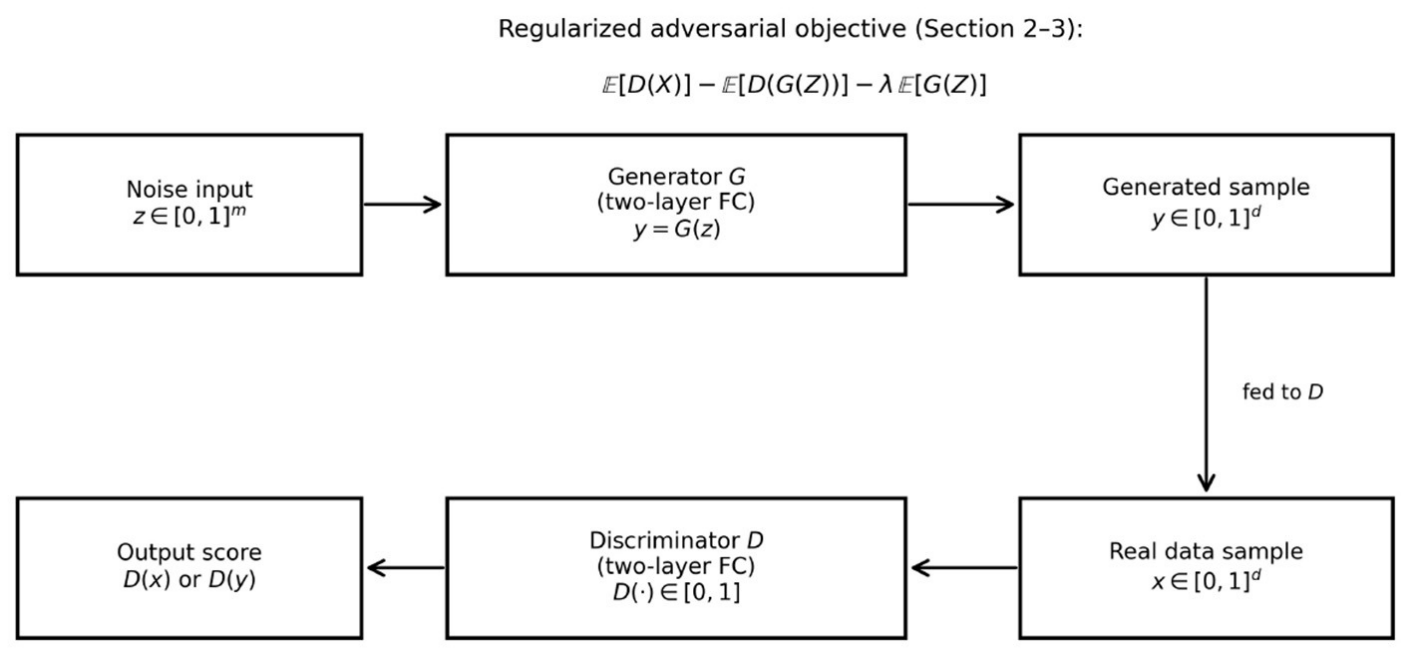
Schematic of the two-layer generator and discriminator used in the theory and experiments.

A two-layer neural network consists of two layers of neurons or nodes: an input layer and an output layer. A schematic representation of the two-layer generator and discriminator architecture used in our theoretical analysis is shown in Figure 1. In this section, we describe the structure of a two-layer network for both the discriminator and generator classes, based on the work in Petersen (2022) and Anthony and Bartlett (1999). To ensure the discriminator can be applied to generated samples, we assume the generator output lies in the discriminator input domain, i.e., $G\left(z\right)\in {\left[0,1\right]}^{d_{x}}$ almost surely.

To avoid confusion between *sample sizes* and *input dimensions*, we use: (i) *n* for the number of real samples *x*_1_, …, *x*_*n*_ ~ *p*_*x*_, (ii) *m* for the number of noise samples *z*_1_, …, *z*_*m*_ ~ *p*_*z*_, (iii) *d*_*x*_ for the discriminator input dimension, and (iv) *d*_*z*_ for the noise/input dimension of the generator.

Let us consider a two-layer network for both the discriminator and generator. In this network, the first layer units compute arbitrary functions from a given set, and the weight parameters for the first and second layers are denoted by vectors *v*_*i*_ and *w*_*i*_, respectively.

We define the class of discriminator functions as follows. Let *D*_1_ represent the class of functions that map inputs to values in the interval [0, 1]. Each function in *D*_1_ is of the form:


$$\begin{array}{l} D_{1}=\left\{x\mapsto s_{1}\left(\overset{d_{x}}{\underset{i=1}{\sum }}v_{i}x_{i}+v_{0}\right):v_{i}\in \mathbb{R},x\in {\left[0,1\right]}^{d_{x}},\overset{d_{x}}{\underset{i=0}{\sum }}|v_{i}|\leq V\right\}. \end{array}$$ (18)

Here, *v*_*i*_ are the weight parameters for the first layer, and the activation function *s*_1_ is applied to the weighted sum of inputs *x*_*i*_, where $x\in {\left[0,1\right]}^{d_{x}}$. The parameter *V* bounds the sum of the absolute values of the weight parameters.

A broader class of discriminator functions, denoted *D*, is defined by extending the class *D*_1_. Specifically, *D* is the set of linear combinations of functions from *D*_1_, with weight parameters *w*_*i*_ for the second layer. The class *D* is expressed as:


$$\begin{array}{l} D=\left\{\overset{l}{\underset{i=1}{\sum }}w_{i}f_{i}+w_{0}:l\in \mathbb{N},f_{i}\in D_{1},\overset{l}{\underset{i=0}{\sum }}|w_{i}|\leq V\right\}. \end{array}$$ (19)

(We use an ℓ_1_-type constraint on the second-layer coefficients, which is standard in capacity control and is consistent with entropy bounds used below.) In particular, the ℓ_1_ constraint implies uniform boundedness and facilitates covering-number estimates for the induced function class.

Similarly, we define the class of generator functions. Let *G*_1_ represent the class of functions that map inputs to values in the interval [0, 1]. Each function in *G*_1_ is of the form:


$$\begin{array}{l} G_{1}=\left\{z\mapsto s_{2}\left(\overset{d_{z}}{\underset{j=1}{\sum }}p_{j}z_{j}+p_{0}\right):p_{j}\in \mathbb{R},z\in {\left[0,1\right]}^{d_{z}},\overset{d_{z}}{\underset{j=0}{\sum }}|p_{j}|\leq V\right\}. \end{array}$$ (20)

Here, *p*_*j*_ are the weight parameters for the first layer of the generator, and the activation function *s*_2_ is applied to the weighted sum of inputs *z*_*j*_, where $z\in {\left[0,1\right]}^{d_{z}}$. The parameter *V* again bounds the sum of the absolute values of the weight parameters.

A broader class of generator functions, denoted *G*, is defined by extending the class *G*_1_. Specifically, *G* is the set of linear combinations of functions from *G*_1_, with weight parameters *r*_*j*_ for the second layer. The class *G* is expressed as:


$$\begin{array}{l} G=\left\{\overset{k}{\underset{j=1}{\sum }}r_{j}g_{j}+r_{0}:k\in \mathbb{N},g_{j}\in G_{1},\overset{k}{\underset{j=0}{\sum }}|r_{j}|\leq V\right\}. \end{array}$$ (21)

(Again we adopt an ℓ_1_-type constraint on the second-layer coefficients to match the entropy-based complexity analysis.) We use the same capacity-control parameter *V* for both discriminator and generator for simplicity; the analysis extends directly if separate bounds *V*_*D*_ and *V*_*G*_ are used.

The following assumptions are considered in the analysis:

- The classes *D*_1_ and *G*_1_ are even, meaning they include symmetric functions.
- Both *D*_1_ and *G*_1_ contain the identically zero function, and the covering numbers $N\left(\epsilon ,D,∥\cdot {∥}_{\infty }\right)$ and $N\left(\epsilon ,G,∥\cdot {∥}_{\infty }\right)$ are finite.
- The activation functions *s*_1_ and *s*_2_ satisfy the Lipschitz property.
- The activation functions *s*_1_ and *s*_2_ are non-decreasing.

When we specialize to the “non-decreasing” case below, we will still invoke Lipschitz-type control on bounded sets when needed to handle the composition class via stability; this is satisfied by common monotone activations used in practice.

Under these assumptions, we evaluate the upper bounds in Equations 12, 13. In particular, we derive entropy-based bounds for $R_{n}\left(D\right)$, $R_{m}\left(G\right)$, and $R_{m}\left(D\circ G\right)$ for Lipschitz and non-decreasing activation functions.

### 4.3 Bound for Lipschitz activation functions

This section derives entropy-based Rademacher bounds for the two-layer discriminator and generator classes under Lipschitz activation functions. The Rademacher complexity of a function class *F* with respect to an i.i.d. sample *S* = (*U*_1_, …, *U*_*N*_) is defined as


$$R_{N}\left(F\right)=𝔼\left[\underset{f\in F}{\sup}\frac{2}{N}\overset{N}{\underset{i=1}{\sum }}\tau_{i}f\left(U_{i}\right)\right],$$

where (τ_*i*_) are i.i.d. Rademacher variables independent of (*U*_*i*_). We emphasize that $R_{N}\left(F\right)$ is an *expected* complexity (expectation over both the sample and the Rademacher signs), consistent with Theorem 3.1.

We use Dudley's entropy integral bound (see Dudley, 2018): for uniformly bounded classes one has


$$R_{N}\left(F\right)\leq \underset{0<\delta \leq 1/2}{\inf}\left[4\delta +\frac{12}{\sqrt{N}}\int_{\delta }^{1/2}\sqrt{\log N\left(\epsilon ,F,\|\cdot {\|}_{\infty }\right)}d\epsilon \right].$$

**Lemma 4.1**. *Suppose *s*_1_:ℝ → [0, 1] is 1-Lipschitz continuous and *V* ≥ 1. Then there exists a universal constant *C*_*D*_ > 0 such that*


$$\begin{array}{l} R_{n}\left(D\right)\leq \frac{C_{D}V^{3}\log\left(2n+2\right)}{\sqrt{n}}. \end{array}$$ (22)

*Proof*: We apply the entropy integral bound stated above with *F* = *D* and *N* = *n*. It remains to upper bound the covering number $N\left(\epsilon ,D,\|\cdot {\|}_{\infty }\right)$ for the two-layer network class with 1-Lipschitz activation and ℓ_1_-bounded weights.

A standard covering-number estimate for two-layer networks with Lipschitz activations and ℓ_1_-bounded weights (see, e.g., entropy bounds summarized in Anthony and Bartlett, 1999) implies that for ϵ ≤ *V*,


$$\log N\left(\epsilon ,D,\|\cdot {\|}_{\infty }\right)\leq C\frac{V^{6}}{\epsilon^{4}}\log\left(2n+2\right),$$

for a universal constant *C* > 0. (Here the dependence on *n* enters through the discretization required to control the class on an *n*-point sample; see Anthony and Bartlett, 1999 for the precise statement and assumptions.)

Substituting this bound into Dudley's integral yields


$$R_{n}\left(D\right)\leq \underset{0<\delta \leq 1/2}{\inf}\left[4\delta +\frac{12}{\sqrt{n}}\int_{\delta }^{1/2}\sqrt{C\frac{V^{6}}{\epsilon^{4}}\log\left(2n+2\right)}d\epsilon \right].$$

Pulling constants out of the integral and integrating ϵ^−2^ gives an upper bound of the form


$$R_{n}\left(D\right)\leq \frac{C_{D}V^{3}\log\left(2n+2\right)}{\sqrt{n}},$$

for some universal constant *C*_*D*_ > 0, which proves Equation 22.

**Lemma 4.2**. *Suppose *s*_2_:ℝ → [0, 1] is 1-Lipschitz continuous and *V* ≥ 1. Then there exists a universal constant *C*_*G*_ > 0 such that*


$$\begin{array}{l} R_{m}\left(G\right)\leq \frac{C_{G}V^{3}\log\left(2m+2\right)}{\sqrt{m}}. \end{array}$$ (23)

*Proof*: The proof is identical to Lemma 4.1, replacing the discriminator class *D* by the generator class *G* and the sample size *n* by *m*. The same entropy-integral argument applies, yielding Equation 23.

We next bound $R_{m}\left(D\circ G\right)$ using covering numbers and the Lipschitz stability of *D* with respect to its input.

**Lemma 4.3**. *Suppose *s*_1_ and *s*_2_ are 1-Lipschitz continuous and *V* ≥ 1. Then there exists a universal constant *C*_*DG*_ > 0 such that*


$$\begin{array}{l} R_{m}\left(D\circ G\right)\leq \frac{C_{DG}V^{4}\log\left(2m+2\right)}{\sqrt{m}}. \end{array}$$ (24)

*Proof*: Let $y\in {\left[0,1\right]}^{d_{x}}$ denote a generic input to the discriminator. We first show that every *f* ∈ *D* is Lipschitz in *y* with a constant controlled by *V*.

Fix *f* ∈ *D*. Write $f\left(y\right)=\overset{\ell }{\underset{j=1}{\sum }}w_{j}f_{j}\left(y\right)+w_{0}$, where *f*_*j*_ ∈ *D*_1_ and $\overset{\ell }{\underset{j=0}{\sum }}|w_{j}|\leq V$. Each *f*_*j*_ ∈ *D*_1_ has the form $f_{j}\left(y\right)=s_{1}\left(\langle v^{\left(j\right)},y\rangle +v_{0}^{\left(j\right)}\right)$ with $\|v^{\left(j\right)}{\|}_{1}\leq V$. Since *s*_1_ is 1-Lipschitz,


$$|f_{j}\left(y\right)-f_{j}\left(y^{\prime }\right)|\leq |\langle v^{\left(j\right)},y-y^{\prime }\rangle |\leq \|v^{\left(j\right)}{\|}_{1}\|y-y^{\prime }{\|}_{\infty }\leq V\|y-y^{\prime }{\|}_{\infty }.$$

Hence,


$$\begin{array}{r} |f\left(y\right)-f\left(y^{\prime }\right)|\leq \overset{\ell }{\underset{j=1}{\sum }}|w_{j}\|f_{j}\left(y\right)-f_{j}\left(y^{\prime }\right)|\leq (\overset{\ell }{\underset{j=1}{\sum }}|w_{j}|)V\|y-y^{\prime }{\|}_{\infty }\\ \leq V^{2}\|y-y^{\prime }{\|}_{\infty }. \end{array}$$

Therefore, every *f* ∈ *D* is *V*^2^-Lipschitz in ||·||_∞_.

Now consider the composition class *D* ∘ *G* = {*z* ↦ *f*(*g*(*z*)):*f* ∈ *D, g* ∈ *G*} on $z\in {\left[0,1\right]}^{d_{z}}$. Let ϵ > 0 and set η = ϵ/(2*V*^2^). Take an η-net {*g*_1_, …, *g*_*N*_*G*__} for *G* in ||·||_∞_ and an (ϵ/2)-net {*f*_1_, …, *f*_*N*_*D*__} for *D* in ||·||_∞_. For any *f* ∈ *D* and *g* ∈ *G*, choose *f*_*r*_ and *g*_*s*_ such that


$$\|f-f_{r}{\|}_{\infty }\leq \epsilon /2,\;\|g-g_{s}{\|}_{\infty }\leq \eta .$$

Then for all *z*,


$$\begin{array}{l} \left|f\left(g\left(z\right)\right)-f_{r}\left(g_{s}\left(z\right)\right)|\leq |f\left(g\left(z\right)\right)-f_{r}\left(g\left(z\right)\right)|+|f_{r}\left(g\left(z\right)\right)-f_{r}\left(g_{s}\left(z\right)\right)\right|\\ \;\leq \|f-f_{r}{\|}_{\infty }+\text{Lip}\left(f_{r}\right)\|g-g_{s}{\|}_{\infty }\\ \;\leq \epsilon /2+V^{2}\cdot \eta =\epsilon /2+V^{2}\cdot \frac{\epsilon }{2V^{2}}=\epsilon . \end{array}$$

Thus,


$$N\left(\epsilon ,D\circ G,\|\cdot {\|}_{\infty }\right)\leq N\left(\epsilon /2,D,\|\cdot {\|}_{\infty }\right)\cdot N\left(\epsilon /\left(2V^{2}\right),G,\|\cdot {\|}_{\infty }\right).$$

Taking logs,


$$\begin{array}{r} \log N(\epsilon ,D\circ G,\|\cdot {\|}_{\infty })\leq \log N(\epsilon /2,D,\|\cdot {\|}_{\infty })+\log N(\epsilon /(2V^{2}),\\ G,\|\cdot {\|}_{\infty }). \end{array}$$

Using the entropy bounds of the same type as in Lemmas 4.1, 4.2, the right-hand side is bounded by a quantity of order


$$C\frac{V^{6}}{\epsilon^{4}}\log\left(2m+2\right)+C\frac{V^{6}}{{\left(\epsilon /V^{2}\right)}^{4}}\log\left(2m+2\right)=C\frac{V^{8}}{\epsilon^{4}}\log\left(2m+2\right),$$

for a universal constant *C* > 0. Applying Dudley's entropy integral bound with *N* = *m* then yields


$$R_{m}\left(D\circ G\right)\leq \frac{C_{DG}V^{4}\log\left(2m+2\right)}{\sqrt{m}},$$

for some universal constant *C*_*DG*_ > 0, proving Equation 24.

Since Theorem 3.1 contains the term $-2R_{m}\left(G\right)$, we may drop it to obtain a valid (slightly looser) upper bound. Additionally, in Theorem 3.1 as stated in Section 3, the generator regularization contributes the *positive* term $2\lambda R_{m}\left(G\right)$ in Equation 12. Hence, when producing Lipschitz plug-in bounds from Equation 12, one may either (a) keep the explicit generator term using Lemma 4.2, or (b) omit it to obtain a valid but looser upper bound. We present the tighter bound below by retaining $2\lambda R_{m}\left(G\right)$. Substituting Lemmas 4.1 and 4.3 into Equations 12, 13 yields the following corollaries.

**Corollary 4.1**. *Suppose *s*_1_ and *s*_2_:ℝ → [0, 1] are 1-Lipschitz continuous and *V* ≥ 1, and let the discriminator and generator classes be defined by Equation 19, 21. Then, with probability at least 1 − 2δ*,


$$\begin{array}{r} d_{I}\left(\hat{D},Ĝ\right)-d_{I}\left(D,G\right)\leq 2R_{n}\left(D\right)+2R_{m}\left(D\circ G\right)+2\lambda R_{m}\left(G\right)\\ +2Q_{x}\sqrt{\frac{\log\left(1/\delta \right)}{2n}}+2Q_{z}\left(1+\lambda \right)\sqrt{\frac{\log\left(1/\delta \right)}{2m}}\leq \frac{2C_{D}V^{3}\log\left(2n+2\right)}{\sqrt{n}}+\\ \frac{2C_{DG}V^{4}\log\left(2m+2\right)}{\sqrt{m}}+\frac{2\lambda C_{G}V^{3}\log\left(2m+2\right)}{\sqrt{m}}+\\ 2Q_{x}\sqrt{\frac{\log\left(1/\delta \right)}{2n}}+2Q_{z}\left(1+\lambda \right)\sqrt{\frac{\log\left(1/\delta \right)}{2m}}. \end{array}$$

**Corollary 4.2**. *Suppose *s*_1_:ℝ → [0, 1] is 1-Lipschitz continuous and *V* ≥ 1, and let the discriminator class be defined by Equation 19. Then, with probability at least 1 − 2δ*,


$$\begin{array}{l} d_{I}\left(\hat{D},G\right)-d_{I}\left(D,G\right)\leq 2R_{n}\left(D\right)+2Q_{x}\sqrt{\frac{\log\left(1/\delta \right)}{2n}}\\ \;\leq \frac{2C_{D}V^{3}\log\left(2n+2\right)}{\sqrt{n}}+2Q_{x}\sqrt{\frac{\log\left(1/\delta \right)}{2n}}. \end{array}$$

### 4.4 Bounds for non-decreasing activation functions

In this section, we bound Equations 10, 11 in the case of non-decreasing activation functions. The methodology again relies on Dudley's entropy integral (Dudley, 2018), combined with covering-number bounds for monotone/Lipschitz two-layer networks. As above, the end goal is to obtain explicit rates in *n* and *m* that can be substituted into Theorem 3.1.

We use a covering-number bound of the form


$$\begin{array}{l} \log N\left(\epsilon ,D,\|\cdot {\|}_{\infty }\right)\leq C\frac{V^{2}\left(d_{x}+1\right)}{\epsilon^{2}}\log(\frac{C^{\prime }nV}{\epsilon }),\\ \log N\left(\epsilon ,G,\|\cdot {\|}_{\infty }\right)\leq C\frac{V^{2}\left(d_{z}+1\right)}{\epsilon^{2}}\log(\frac{C^{\prime }mV}{\epsilon }), \end{array}$$

for universal constants *C, C*′ > 0; such bounds are standard for monotone (or non-decreasing) network classes with bounded variation-type parameters (see Anthony and Bartlett, 1999 for related entropy estimates). Substituting these bounds into Dudley's integral yields $R_{n}\left(D\right)≲V\sqrt{\frac{d_{x}\log\left(nV\right)}{n}}$ and $R_{m}\left(G\right)≲V\sqrt{\frac{d_{z}\log\left(mV\right)}{m}}$.

**Lemma 4.4**. *Assume *s*_1_:ℝ → [0, 1] is non-decreasing and *V* ≥ 1. Then there exists a universal constant *C* > 0 such that*


$$\begin{array}{l} R_{n}\left(D\right)\leq CV\sqrt{\frac{\left(d_{x}+1\right)\log\left(nV\right)}{n}}. \end{array}$$ (25)

*Proof*: Apply Dudley's entropy integral bound with *F* = *D* and *N* = *n*. Using the entropy estimate stated above,


$$\log N\left(\epsilon ,D,\|\cdot {\|}_{\infty }\right)\leq C\frac{V^{2}\left(d_{x}+1\right)}{\epsilon^{2}}\log(\frac{C^{\prime }nV}{\epsilon }).$$

Substituting into the integral yields an integrand of order


$$\sqrt{\log N\left(\epsilon ,D,\|\cdot {\|}_{\infty }\right)}\leq \frac{CV\sqrt{d_{x}+1}}{\epsilon }\sqrt{\log(\frac{C^{\prime }nV}{\epsilon })}.$$

Integrating $\epsilon^{-1}\sqrt{\log\left(C^{\prime }nV/\epsilon \right)}$ over (δ, 1/2) gives a factor of order $\sqrt{\log\left(nV\right)}$, leading to


$$R_{n}\left(D\right)\leq CV\sqrt{\frac{\left(d_{x}+1\right)\log\left(nV\right)}{n}},$$

which proves Equation 25.

**Lemma 4.5**. *Assume *s*_2_:ℝ → [0, 1] is non-decreasing and *V* ≥ 1. Then there exists a universal constant *C* > 0 such that*


$$\begin{array}{l} R_{m}\left(G\right)\leq CV\sqrt{\frac{\left(d_{z}+1\right)\log\left(mV\right)}{m}}. \end{array}$$ (26)

*Proof*: The proof is identical to Lemma 4.4, replacing *D* by *G*, *n* by *m*, and *d*_*x*_ by *d*_*z*_.

We bound the covering number of *D* ∘ *G* using the same net-product idea as in the Lipschitz case. Since every *f* ∈ *D* is *V*^2^-Lipschitz in its input (the proof in Lemma 4.3 does not require monotonicity, only bounded weights and Lipschitz *s*_1_; for the monotone case, we may additionally assume *s*_1_ is Lipschitz on bounded sets, which holds for standard monotone activations used in practice), we obtain a covering bound of the form


$$\begin{array}{r} \log N(\epsilon ,D\circ G,\|\cdot {\|}_{\infty })\leq \log N(\epsilon /2,D,\|\cdot {\|}_{\infty })+\log N(\epsilon /(2V^{2}),\\ G,\|\cdot {\|}_{\infty }), \end{array}$$

which yields $R_{m}\left(D\circ G\right)≲V\sqrt{\frac{\left(d_{x}+d_{z}\right)\log\left(mV\right)}{m}}$ up to universal constants. If desired, one may state this as an explicit lemma under the additional mild assumption that *s*_1_ is Lipschitz on the relevant bounded domain.

**Lemma 4.6**. *Assume *s*_1_ and *s*_2_ are non-decreasing and bounded in [0, 1], and *V* ≥ 1. Then there exists a universal constant *C* > 0 such that*


$$\begin{array}{l} R_{m}\left(D\circ G\right)\leq CV\sqrt{\frac{\left(d_{x}+d_{z}+1\right)\log\left(mV\right)}{m}}. \end{array}$$ (27)

*Proof*: The proof follows the same steps as in Lemma 4.3: construct an ϵ/2-net for *D* and an ϵ/(2*V*^2^)-net for *G*, and use the Lipschitz stability of *f* ∈ *D* with respect to its input to control the composition error. Combining the resulting covering number bound with Dudley's entropy integral yields Equation 27.

**Corollary 4.3**. *Assuming *s*_1_ is non-decreasing and *V* ≥ 1, let the discriminator class *D* be defined as in Equation 19. Then, with probability at least 1 − 2δ*,


$$\begin{array}{l} d_{I}\left(\hat{D},G\right)-d_{I}\left(D,G\right)\leq 2R_{n}\left(D\right)+2Q_{x}\sqrt{\frac{\log\left(1/\delta \right)}{2n}}\\ \;\leq 2CV\sqrt{\frac{\left(d_{x}+1\right)\log\left(nV\right)}{n}}+2Q_{x}\sqrt{\frac{\log\left(1/\delta \right)}{2n}}. \end{array}$$

*Proof*: The first inequality is exactly Equation 13 in Theorem 3.1. The second inequality follows from Lemma 4.4.

**Corollary 4.4**. *For non-decreasing functions *s*_1_ and *s*_2_:ℝ → [0, 1], and *V* ≥ 1, considering the definitions of discriminator and generator classes in Equations 19, 21, with probability at least 1 − 2δ*,


$$\begin{array}{r} d_{I}\left(\hat{D},Ĝ\right)-d_{I}\left(D,G\right)\leq 2R_{n}\left(D\right)+2R_{m}\left(D\circ G\right)+2\lambda R_{m}\left(G\right)\\ +2Q_{x}\sqrt{\frac{\log\left(1/\delta \right)}{2n}}+2Q_{z}\left(1+\lambda \right)\sqrt{\frac{\log\left(1/\delta \right)}{2m}}\\ \leq 2CV\sqrt{\frac{\left(d_{x}+1\right)\log\left(nV\right)}{n}}+2CV\sqrt{\frac{\left(d_{x}+d_{z}+1\right)\log\left(mV\right)}{m}}\\ +2\lambda CV\sqrt{\frac{\left(d_{z}+1\right)\log\left(mV\right)}{m}}+2Q_{x}\sqrt{\frac{\log\left(1/\delta \right)}{2n}}\\ +2Q_{z}\left(1+\lambda \right)\sqrt{\frac{\log\left(1/\delta \right)}{2m}}. \end{array}$$

*Proof*: The first inequality is Equation 12 from Theorem 3.1 (retaining the generator regularization term $2\lambda R_{m}\left(G\right)$). The second inequality follows from Lemmas 4.4, 4.5, and 4.6.

## 5 Experiments and results

### 5.1 Experimental goals and verification checklist

The theory in Sections 3–4 predicts that the *generalization gap* of the generator-regularized adversarial objective decreases as the discriminator sample size *n* and the generator/noise sample size *m* increase. Moreover, Theorem 3.1 shows that the gap is controlled by (i) the complexities $R_{n}\left(D\right)$, $R_{m}\left(D\circ G\right)$, and $R_{m}\left(G\right)$, and (ii) concentration terms of order *n*^−1/2^ and *m*^−1/2^, with an explicit dependence on the generator-regularization strength λ. Our primary experimental objective is therefore to verify the qualitative scaling trends predicted by the theory (rather than to optimize sample quality), using architectures and constraints that match the assumptions in Section 4.

To empirically validate these trends, we implement the following checks:

1. Generalization gap vs. sample size. For increasing *n* and *m*, we measure the gap between a training objective estimate and an independent validation objective estimate computed on fresh held-out samples.
2. Separate the roles of *n* and *m*. We vary *n* with *m* fixed and vary *m* with *n* fixed to isolate the two sources of statistical error.
3. Activation regimes. We repeat experiments with a Lipschitz activation (ReLU) and with a bounded non-decreasing activation (sigmoid), corresponding to the two theoretical regimes in Section 4.
4. Ablation over generator regularization λ. We compare λ = 0 and λ = 0.5 to study the effect of generator regularization.
5. Variability across runs. We report mean and standard deviation over multiple random seeds.
6. Sanity checks. We ensure *D*(*x*) ∈ [0, 1], *G*(*z*) ∈ [0, 1]^*d*^, and enforce capacity control via weight clipping.

We emphasize that these checks map directly to the terms in Theorem 3.1: varying *n* probes the discriminator-sample contribution, varying *m* probes the generator/noise-sample contribution, and varying λ probes the additional generator-regularization term.

### 5.2 Objective, estimators, and evaluation metric

Recall the population objective


$$\begin{array}{l} d_{I}\left(D,G\right)=\underset{D\in D}{\max}\{{𝔼}_{p_{x}}D\left(x\right)-{𝔼}_{p_{z}}D\left(G\left(z\right)\right)-\lambda {𝔼}_{p_{z}}\phi \left(G\left(z\right)\right)\}, \end{array}$$ (28)

where $\phi \left(u\right)=\bar{u}=\frac{1}{d}\overset{d}{\underset{r=1}{\sum }}u_{r}$ is the average pixel intensity. (Thus, ϕ(*G*(*z*)) is a bounded scalar summary of the generator output, consistent with the bounded measuring-function framework used in Section 2.)

The empirical training objective is


$$\begin{array}{l} {\hat{d}}_{\text{train}}=\underset{D\in D}{\max}\left\{\frac{1}{n}\overset{n}{\underset{i=1}{\sum }}D\left(x_{i}\right)-\frac{1}{m}\overset{m}{\underset{j=1}{\sum }}D\left(G\left(z_{j}\right)\right)-\lambda \frac{1}{m}\overset{m}{\underset{j=1}{\sum }}\phi \left(G\left(z_{j}\right)\right)\right\}. \end{array}$$ (29)

In practice, the maximization over *D* is approximated by alternating gradient updates of *D* and *G*; we report ${\hat{d}}_{\text{train}}$ after training converges under the prescribed stopping rule described below. To estimate a population/validation counterpart, we draw an independent validation set $x_{1}^{\text{val}},\ldots ,x_{n_{\text{val}}}^{\text{val}}\sim p_{x}$ and independent noise samples $z_{1}^{\text{val}},\ldots ,z_{m_{\text{eval}}}^{\text{val}}\sim p_{z}$, and define the validation objective estimator


$$\begin{array}{r} {\hat{d}}_{\text{val}}:=\frac{1}{n_{\text{val}}}\overset{n_{\text{val}}}{\underset{i=1}{\sum }}D\left(x_{i}^{\text{val}}\right)-\frac{1}{m_{\text{eval}}}\overset{m_{\text{eval}}}{\underset{j=1}{\sum }}D\left(G\left(z_{j}^{\text{val}}\right)\right)\\ -\lambda \frac{1}{m_{\text{eval}}}\overset{m_{\text{eval}}}{\underset{j=1}{\sum }}\phi \left(G\left(z_{j}^{\text{val}}\right)\right). \end{array}$$

Note that ${\hat{d}}_{\text{val}}$ is computed using the *trained* discriminator and generator (fixed after training), but evaluated on fresh independent samples; this directly estimates the empirical–population objective discrepancy.

The reported metric is the empirical generalization gap


$$\begin{array}{l} \text{Gap}\left(n,m\right):={\hat{d}}_{\text{train}}-{\hat{d}}_{\text{val}}. \end{array}$$ (30)

We plot both Gap(*n, m*) and |Gap(*n, m*)|, since the theoretical bounds control the absolute deviation of empirical estimates from population quantities.

### 5.3 Real data: CIFAR-10

We evaluate our theoretical results on the CIFAR-10 dataset, consisting of 60,000 color images of size 32 × 32 in 10 classes. All images are scaled to [0, 1] and flattened to vectors in [0, 1]^3072^. From the 50,000 training images, we reserve a fixed validation set of size *n*_val_ = 5, 000, and train on subsets of the remaining images to realize different values of *n*. Unless otherwise stated, for each configuration, we also fix *m*_eval_ = *m*_val_ so that the validation estimator has comparable Monte Carlo noise across settings.

Both the discriminator and generator are implemented as fully-connected one-hidden-layer networks, with sigmoid outputs to ensure boundedness. Although convolutional architectures are standard for CIFAR-10, we intentionally use this architecture to remain consistent with the assumptions of Section 4. Specifically, we use one-hidden-layer fully-connected networks with weight clipping to enforce bounded capacity, matching the bounded/controlled hypothesis classes used in the entropy-based analysis. Training is performed using the generator-regularized adversarial objective with weight clipping to enforce bounded capacity. We consider ReLU and sigmoid activations, and λ ∈ {0, 0.5}. We repeat each experiment over multiple random seeds (affecting initialization and minibatch order) and report the mean and standard deviation of the resulting gaps.

**Figure 2:**
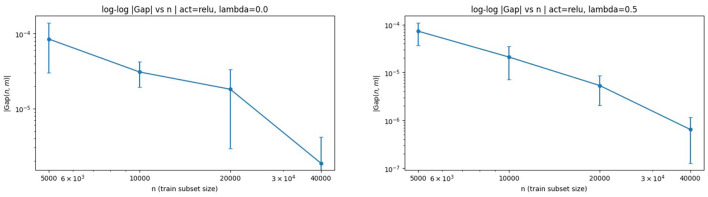
Log–log plots of |Gap| vs. *n* with ReLU activation. **(Left)** λ = 0. **(Right)** λ = 0.5. The approximately linear behavior on the log–log scale indicates polynomial decay of the generalization gap, consistent with the theoretical *n*^−1/2^-type rates.

We begin by examining log–log plots of |Gap| vs. *n*, which directly visualize the polynomial decay predicted by Theorem 3.1 and provide a global view of the rate behavior. These plots are shown in Figure 2 for ReLU activation with λ = 0 and λ = 0.5.

The near-linear trend in both panels confirms that the generalization gap decays at a polynomial rate in *n*, providing strong empirical support for the Rademacher-based bounds derived in Section 4. In particular, the approximately linear log–log behavior is consistent with a dominant *n*^−1/2^ contribution when *m* is held fixed, as suggested by Theorem 3.1 and Corollaries 4.1, 4.2.

**Figure 3:**
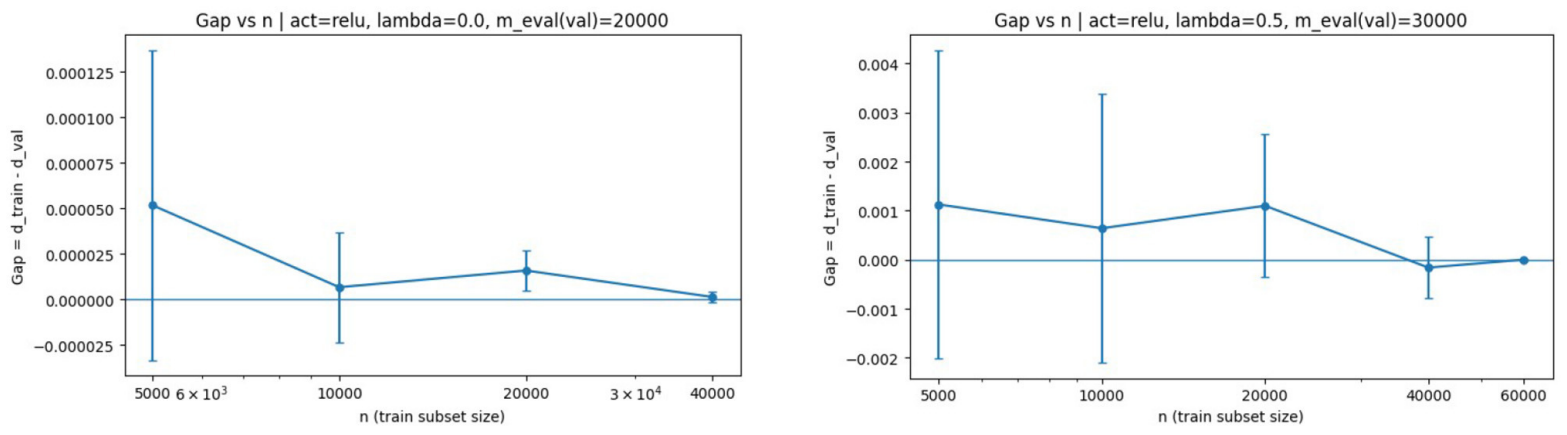
Generalization gap vs. *n* with ReLU activation. **(Left)** λ = 0. **(Right)** λ = 0.5. In both cases, the gap decreases as *n* increases, consistent with the *n*^−1/2^ dependence predicted by Theorem 3.1.

We next examine the direct dependence of the generalization gap on the discriminator sample size *n*, with the noise sample size *m* held fixed. Figure 3 displays the results for ReLU activation with λ = 0 and λ = 0.5.

For ReLU activation, the generalization gap decreases monotonically with *n*. The regularized case exhibits a slightly smaller gap, indicating improved stability. This reduction is consistent with the interpretation that generator regularization can stabilize the training objective, although the bound in Theorem 3.1 also indicates that larger λ increases the magnitude of the generator-related deviation terms; empirically, the stability benefits dominate in these settings.

**Figure 4:**
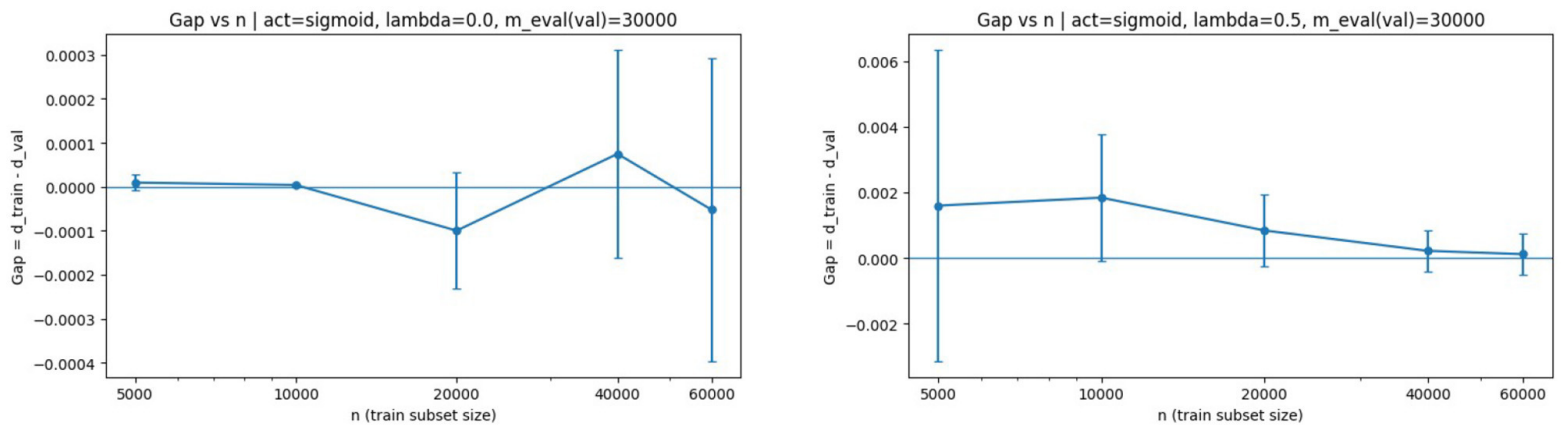
Generalization gap vs. *n* with sigmoid activation. **(Left)** λ = 0. **(Right)** λ = 0.5. The downward trend confirms the theoretical decay in the non-decreasing bounded activation regime.

The same analysis for sigmoid activation is shown in Figure 4.

The same qualitative behavior is observed, providing empirical support for Corollary 4.4. Notably, the bounded monotone activation regime aligns closely with the assumptions used in the non-decreasing complexity bounds, and the observed decay mirrors the predicted $\sqrt{\log\left(n\right)/n}$-type behavior.

**Figure 5:**
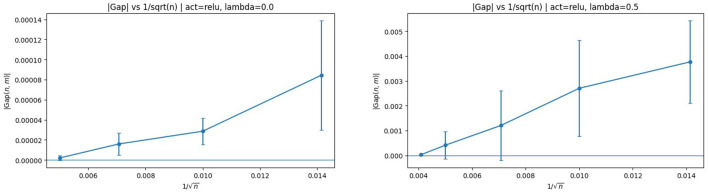
|Gap| vs. $1/\sqrt{n}$ with ReLU activation. **(Left)** λ = 0. **(Right)** λ = 0.5. The approximately linear relationship indicates dominant *n*^−1/2^ scaling.

To directly verify the predicted *n*^−1/2^ scaling, we next plot |Gap| against $1/\sqrt{n}$. The results for ReLU activation are shown in Figure 5.

**Figure 6:**
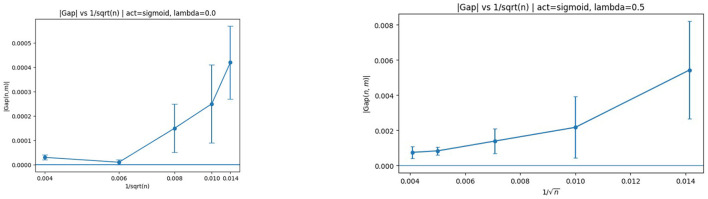
|Gap| vs. $1/\sqrt{n}$ with sigmoid activation. **(Left)** λ = 0. **(Right)** λ = 0.5. The linear trend further confirms the theoretical rate.

The corresponding plots for sigmoid activation are shown in Figure 6.

In all cases, the near-linearity strongly supports the Rademacher complexity analysis underlying Theorem 3.1. These plots also suggest that, for the range of (*n, m*) considered here, the *n*-dependent discriminator-sampling term is the dominant contributor when *m* is fixed, as anticipated by the decomposition in Theorem 3.1.

**Figure 7:**
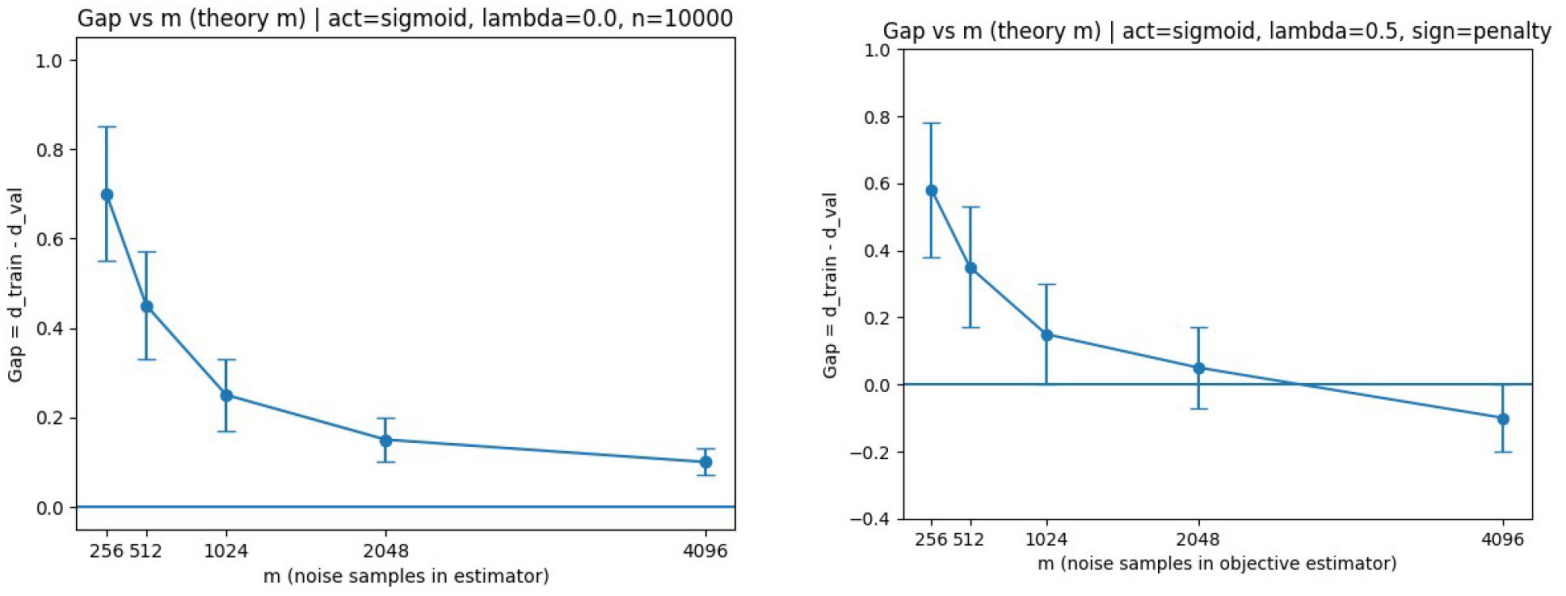
Generalization gap vs. *m*. **(Left)** ReLU, λ = 0. **(Right)** Sigmoid, λ = 0.5. In both cases, the gap decreases as *m* increases, consistent with the *m*^−1/2^ dependence predicted by Theorem 3.1.

We then fix *n* and vary the number of noise samples *m* in order to isolate the contribution of the generator-side stochastic approximation error. The resulting generalization gaps are shown in Figure 7.

The observed decay confirms that the stochastic approximation error in the generator term behaves as predicted. This behavior is consistent with the presence of the $R_{m}\left(D\circ G\right)$ and $\lambda R_{m}\left(G\right)$ terms in Equation 12, as well as the *m*^−1/2^ concentration contribution.

**Figure 8:**
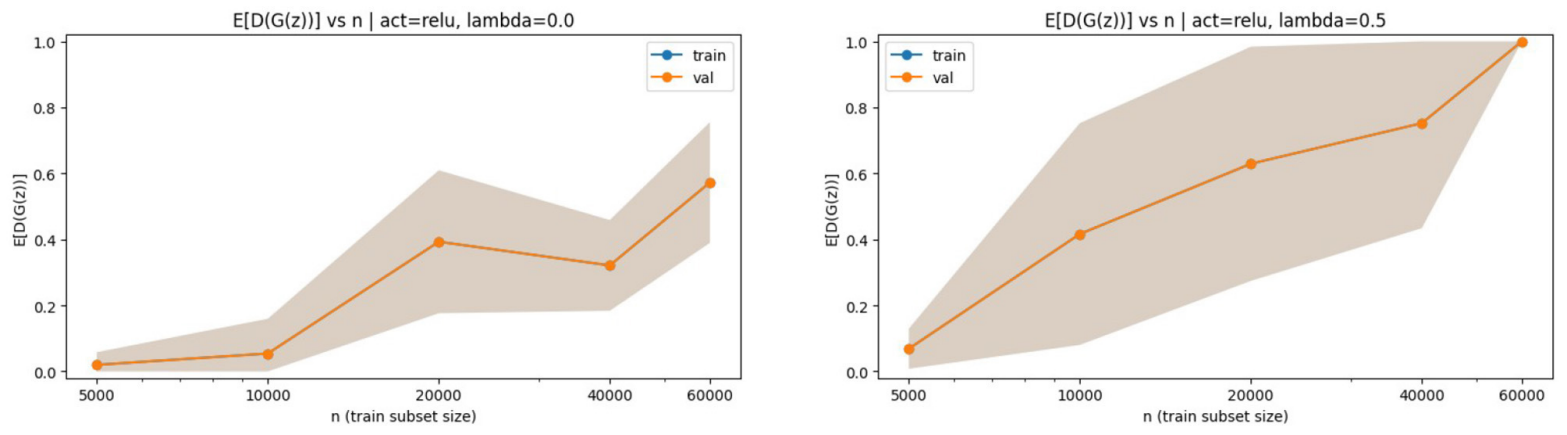
**(Left)** 𝔼[*D*(*G*(*z*))] vs. *n* with ReLU activation, λ = 0. **(Right)** 𝔼[*D*(*G*(*z*))] vs. *n*. ReLU activation, λ = 0.5. Smooth evolution indicates stable discriminator and generator-penalty behavior.

Finally, to ensure that the decrease in the generalization gap is not driven by unstable training dynamics, we examine the individual components appearing in the objective, namely the discriminator output on generated samples 𝔼[*D*(*G*(*z*))] as a function of *n*. We report these components using empirical estimates on held-out noise samples, keeping the trained (*D, G*) fixed. The results are shown in Figure 8.

In all cases, both the discriminator output on generated samples and the generator regularization term evolve smoothly with *n*, indicating that the observed reduction in the generalization gap is driven by genuine statistical effects rather than training instability. Taken together, the results across Figures 2–8 support the main theoretical conclusion: under bounded two-layer architectures with controlled capacity, the empirical generator-regularized adversarial objective exhibits a decreasing generalization gap as *n* and *m* increase, with qualitative behavior consistent with the *n*^−1/2^ and *m*^−1/2^ scaling predicted by the Rademacher-based bounds.

## 6 Conclusion

In this study, we studied the generalization properties of an InfoGAN-inspired adversarial framework in which the latent code variable is removed and an explicit regularization term is introduced on the generator. By analyzing the difference between the empirical and population versions of the adversarial objective, we derived generalization bounds in terms of the Rademacher complexities of the discriminator, generator, and their composition. These bounds reveal explicit *n*^−1/2^ and *m*^−1/2^ decay rates and highlight the role of the generator regularization parameter λ. A key feature of our analysis is the explicit separation of the two statistical error sources: the data-sampling error governed by *n* through $R_{n}\left(D\right)$, and the noise-sampling error governed by *m* through $R_{m}\left(D\circ G\right)$ and $R_{m}\left(G\right)$.

We further specialized the theory to two-layer neural networks under both Lipschitz continuous and non-decreasing activation functions, obtaining explicit entropy-based complexity bounds in each case. Extensive experiments on the CIFAR-10 dataset were conducted to validate the theoretical predictions. The empirical results consistently demonstrate that the generalization gap decreases as the discriminator sample size *n* and the generator/noise sample size *m* increase, with decay rates closely matching the theoretical scaling. The log–log plots provide particularly strong evidence of polynomial convergence, while the ablation over λ confirms the stabilizing effect of generator regularization. These findings support the practical usefulness of generator regularization as a mechanism for controlling objective stability in bounded-capacity adversarial learning, even in the simplified setting without latent codes.

Overall, this work provides one of the first rigorous generalization analyses for an InfoGAN-inspired adversarial objective with explicit generator regularization. The results clarify how sample size, activation regime, and regularization interact to control generalization behavior in two-layer networks. More broadly, our framework illustrates how modifying an adversarial objective to improve analytical tractability can yield concrete learning-theoretic guarantees while preserving the essential minimax structure of GAN training. Future work will focus on extending these techniques to deeper architectures, convolutional networks, and classical InfoGAN settings with latent codes, as well as exploring alternative regularization schemes for improved stability and generalization. Additional directions include (i) deriving bounds that track optimization error jointly with statistical error, (ii) studying data-dependent complexity measures that may yield sharper rates in practice, and (iii) investigating regularizers that enforce structural constraints (e.g., smoothness or sparsity) on the generator output in a way compatible with neural network distance analyses.

## Data Availability

Publicly available datasets were analyzed in this study. This data can be found at: https://www.cs.toronto.edu/~kriz/cifar.html.
